# Supplementary material for: Progressing Towards the Sustainable Development of Cream Formulations
Source: Pharmaceutics. 2020 Jul 9;12(7):647. doi: 10.3390/pharmaceutics12070647 (PMC7407566; doi:10.3390/pharmaceutics12070647)
Supplement: Supplementary file 1 [file pharmaceutics-12-00647-s001.pdf]

**Supplementary material**

# Progressing Towards the Sustainable Development of Cream Formulations

**Ana Simões [1,2], Francisco Veiga [1,2] and Carla Vitorino [1,3,4,*]**

[1] Faculty of Pharmacy, University of Coimbra, 3000-548 Coimbra, Portugal; simoesana88@gmail.com (A.S.); fveiga@ff.uc.pt (F.V.)
[2] Associated Laboratory for Green Chemistry of the Network of Chemistry and Technology (LAQV/REQUIMTE), Group of Pharmaceutical Technology, Faculty of Pharmacy, University of Coimbra, 3000-548 Coimbra, Portugal
[3] Coimbra Chemistry Center, Department of Chemistry, University of Coimbra, 3004-535 Coimbra, Portugal
[4] Centre for Neurosciences and Cell Biology (CNC), Faculty of Medicine, University of Coimbra, 3004-504 Coimbra, Portugal
* Correspondence: csvitorino@ff.uc.pt; Tel.: +351-239-488-400

**Table S1.** ANOVA parameter summary of fitted model's characterization.

| CQAs | Regression | | | Lack of fit | |
|---|---|---|---|---|---|
| | $F_1$ | Prob > F | $R^2$ | $F_2$ | Prob > F |
| Droplet size | 12.9885 | 0.0057** | 0.958982 | 2.5973 | 0.2902 |
| $\eta_{10}$ | 29.8836 | 0.0008** | 0.981749 | 1.5428 | 0.4165 |
| $S_R$ | 14.9581 | 0.0041** | 0.964189 | 6.1523 | 0.143 |
| LVR plateau | 7.8369 | 0.0177* | 0.933803 | 19.232 | 0.0498* |
| $\tau_0$ | 5.2838 | 0.0407* | 0.90486 | 1.9635 | 0.355 |
| $\tau_f$ | 3.4703 | 0.0921 | 0.862004 | 23.756 | 0.0407* |
| $G`$ | 6.9352 | 0.0231* | 0.925835 | 32.4403 | 0.0301* |
| $G``$ | 5.6682 | 0.0353* | 0.910736 | 35.2677 | 0.0277* |
| $tan\ \delta$ | 2.4786 | 0.1651 | 0.816897 | 2.3963 | 0.308 |
| $c_1$ | 0.9974 | 0.5317 | 0.642256 | 5.1618 | 0.166 |
| $c_2$ | 2.4233 | 0.1712 | 0.813503 | 6.8294 | 0.1340 |
| $R_{6h}$ | 0.5377 | 0.8027 | 0.49183 | 5.0926 | 0.1685 |
| $R_{24h}$ | 3.0795 | 0.1142 | 0.847167 | 3.8964 | 0.2109 |
| $J_{ss}$ | 1.2724 | 0.4151 | 0.696082 | 0.0308 | 0.9907 |
| $K_p$ | 1.064 | 0.5004 | 0.65696 | 0.0406 | 0.9863 |
| $Q_{6h}$ | 0.5377 | 0.8027 | 0.49183 | 5.0926 | 0.1685 |
| $Q_{24h}$ | 0.6115 | 0.7545 | 0.523969 | 0.1184 | 0.9415 |
| $Q_{48h}$ | 0.7798 | 0.6494 | 0.583961 | 0.0439 | 0.9846 |
| Assay | 2.5771 | 0.1548 | 0.822655 | 22.0697 | 0.0437* |
| pH | 6.7825 | 0.0242* | 0.924291 | 0.0845 | 0.9623 |
| Instability index | 31.6506 | 0.0007** | 0.98275 | 1.9759 | 0.3534 |
| Sedimentation rate | 1.7489 | 0.279 | 0.758916 | 23.7031 | 0.0408* |
| Creaming rate | 38.8792 | 0.0004** | 0.985912 | 73.7965 | 0.0134* |

Signif. codes: ′***`0.001′**`0.01 ′*`0.05.

**Table S2.** Coefficients values and Student's t-test analysis.

| CQAs | Regression | $\beta_0$ | $\beta_1$ | $\beta_2$ | $\beta_3$ | $\beta_{12}$ | $\beta_{13}$ | $\beta_{23}$ | $\beta_{11}$ | $\beta_{22}$ | $\beta_{33}$ |
|---|---|---|---|---|---|---|---|---|---|---|---|
| Droplet size | **Coefficient value** | 2.59 | 0.195 | -0.027 | -0.566 | 0.095 | 0.158 | -0.246 | 0.0013 | -0.1213 | -0.126 |
| | t Ratio | 26.19 | 3.23 | -0.45 | -9.41 | 1.12 | 1.86 | -2.9 | 0.01 | -1.37 | 26.19 |
| | Prob >\|t\| | **<0.0001*** ** | **0.0233*** | 0.6682 | **0.0002**** | 0.3152 | 0.1216 | **0.0339*** | 0.9893 | 0.2294 | **<0.0001*** ** |
| $\eta_{10}$ | **Coefficient value** | 7.127 | 4.382 | 0.075 | 0.358 | 0.32 | 0.8398 | -0.08 | -1.49 | 0.095 | -0.025 |
| | t Ratio | 15.72 | 15.85 | 0.27 | 1.3 | 0.82 | 2.16 | -0.21 | -3.67 | 0.23 | 15.72 |
| | Prob >\|t\| | **<0.0001*** ** | **<0.0001*** ** | 0.7967 | 0.2515 | 0.4493 | 0.0835 | 0.8453 | **0.0144*** | 0.8243 | **<0.0001*** ** |
| $S_R$ | **Coefficient value** | 16490.457 | 33638.394 | 1374.818 | 3949.038 | -1728.925 | -343.890 | 995.626 | 17440.783 | 4531.708 | 16490.457 |
| | t Ratio | 3.23 | 10.79 | 0.44 | 1.27 | -0.39 | -0.08 | 0.23 | 3.81 | 0.99 | 3.23 |
| | Prob >\|t\| | **0.0233*** | **0.0001**** | 0.6776 | 0.2602 | 0.7105 | 0.9406 | 0.8296 | **0.0125*** | 0.3678 | **0.0233*** |
| LVR plateau | **Coefficient value** | 4483.521 | 15139.833 | -469.379 | 1463.244 | -3911.885 | -1055.075 | 2803.487 | 9620.348 | 3279.695 | 4483.5214 |
| | t Ratio | 1.34 | 7.42 | -0.23 | 0.72 | -1.36 | -0.37 | 0.98 | 3.21 | 1.09 | 1.34 |
| | Prob >\|t\| | 0.2375 | **0.0007**** | 0.8271 | 0.5043 | 0.2322 | 0.7284 | 0.3738 | **0.0237*** | 0.3235 | 0.2375 |
| $\tau_0$ | **Coefficient value** | 17.92 | 19.524 | -11.92 | 0.169 | -8.258 | -0.402 | 4.165 | -0.083 | 13.6 | 17.92 |
| | t Ratio | 2.93 | 5.24 | -3.2 | 0.05 | -1.57 | -0.08 | 0.79 | -0.02 | 2.48 | 2.93 |
| | Prob >\|t\| | **0.0325*** | **0.0034**** | **0.024*** | 0.9656 | 0.1772 | 0.9419 | 0.4635 | 0.9884 | 0.0555 | **0.0325*** |
| $\tau_f$ | **Coefficient value** | 23.249 | 35.7 | -10.121 | -1.6 | -0.622 | -4.039 | 6.226 | 9.389 | 22.574 | 23.249 |
| | t Ratio | 1.92 | 4.83 | -1.37 | -0.22 | -0.06 | -0.39 | 0.6 | 0.87 | 2.08 | 1.92 |
| | Prob >\|t\| | 0.1129 | **0.0047**** | 0.2289 | 0.8367 | 0.9547 | 0.7138 | 0.5756 | 0.4264 | 0.092 | 0.1129 |
| *G`* | **Coefficient value** | 4508.099 | 17153.397 | -1032.435 | 1810.603 | -5289.17 | -985.054 | 3239.560 | 11356.588 | 4452.188 | 4508.099 |
| | t Ratio | 1.1 | 6.87 | -0.41 | 0.73 | -1.5 | -0.28 | 0.92 | 3.09 | 1.21 | 1.1 |
| | Prob >\|t\| | 0.3211 | **0.001**** | 0.6965 | 0.5003 | 0.1939 | 0.7907 | 0.3995 | **0.027*** | 0.2792 | 0.3211 |

| | | | | | | | | | | | |
|---|---|---|---|---|---|---|---|---|---|---|---|
| | **Coefficient value** | 1234.326 | 6135.938 | -387.326 | 672.716 | -2222.433 | -479.471 | 1432.262 | 4308.715 | 1234.326 | 6135.938 |
| *G``* | t Ratio | 0.75 | 6.08 | -0.38 | 0.67 | -1.56 | -0.34 | 1.01 | 2.9 | 0.75 | 6.08 |
| | Prob >\|t\| | 0.4893 | **0.0017**** | 0.7171 | 0.534 | 0.1796 | 0.7497 | 0.3601 | **0.0336*** | 0.4893 | **0.0017**** |
| | **Coefficient value** | 0.26480 | 1.21e$^{-03}$ | 6.17e$^{-04}$ | 0.04538 | 0.01425 | -0.06970 | 0.02458 | 0.06825 | 5.00e$^{-04}$ | 0.03945 |
| $tan\ \delta$ | t Ratio | 8.64 | 0.06 | 0.03 | 2.43 | 0.54 | -2.65 | 0.93 | 2.48 | 0.02 | 1.42 |
| | Prob >\|t\| | **0.0003**** | 0.951 | 0.9749 | 0.0593 | 0.6124 | **0.0456*** | 0.3935 | 0.0555 | 0.9862 | 0.2144 |
| | **Coefficient value** | 126.31 | 3.3484 | -7.7206 | 7.1475 | 5.585 | -11.448 | -8.731 | 19.732 | 2.242 | 16.218 |
| $c_1$ | t Ratio | 10.04 | 0.44 | -1.01 | 0.93 | 0.52 | -1.06 | -0.81 | 1.75 | 0.20 | 1.42 |
| | Prob >\|t\| | **0.0002*** | 0.6808 | 0.3606 | 0.3936 | 0.6281 | 0.3380 | 0.4559 | 0.1405 | 0.8502 | 0.2138 |
| | **Coefficient value** | 0.5127 | -0.0146 | 0.0007 | 0.0413 | 0.0055 | -0.0224 | 0.0412 | -0.0003 | 0.0003 | 0.0028 |
| $c_2$ | t Ratio | 26.49 | -1.24 | 0.06 | 3.50 | 0.33 | -1.35 | 2.48 | -0.01 | 0.01 | 0.16 |
| | Prob >\|t\| | **<0.0001***** | 0.2699 | 0.9519 | **0.0173*** | 0.7548 | 0.2351 | 0.056 | 0.9891 | 0.9891 | 0.8805 |
| | **Coefficient value** | 1.76424 | 0.02716 | 0.05 | -0.025 | -0.05 | 0.04753 | -3.46e$^{-18}$ | 0.01667 | -0.03333 | 0.01910 |
| $R_{6h}$ | t Ratio | 28.31 | 0.71 | 1.31 | -0.66 | -0.93 | 0.89 | 0 | 0.3 | -0.6 | 0.34 |
| | Prob >\|t\| | **<.0001***** | 0.507 | 0.2456 | 0.5391 | 0.3942 | 0.4154 | 1 | 0.7774 | 0.5766 | 0.7488 |
| | **Coefficient value** | 10.82357 | -0.78275 | 1.40621 | 1.78875 | 0.56500 | -3.55293 | 0.46671 | 3.74792 | 0.93542 | 3.11101 |
| $R_{24h}$ | t Ratio | 7.27 | -0.86 | 1.55 | 1.97 | 0.44 | -2.78 | 0.37 | 2.81 | 0.7 | 2.31 |
| | Prob >\|t\| | **0.0008*** | 0.428 | 0.1821 | 0.1053 | 0.6776 | **0.039*** | 0.73 | **0.0375*** | 0.5143 | 0.069 |
| | **Coefficient value** | 0.65803 | 0.03875 | -0.02483 | -0.072 | -0.03 | 0.0303 | -0.01002 | -0.13992 | -0.20342 | -0.15087 |
| $J_{ss}$ | t Ratio | 6.58 | 0.63 | -0.41 | -1.18 | -0.35 | 0.35 | -0.12 | -1.56 | -2.27 | -1.67 |
| | Prob >\|t\| | **0.0012**** | 0.5534 | 0.701 | 0.2903 | 0.7418 | 0.7389 | 0.9118 | 0.1794 | 0.0726 | 0.1567 |
| | **Coefficient value** | 0.01725 | 6.36e$^{-04}$ | -3.27e$^{-04}$ | -1.64 e$^{-03}$ | 8.75e$^{-05}$ | 4.56E$^{-04}$ | 1.98E$^{-04}$ | -3.15 e$^{-03}$ | -5.35 e$^{-03}$ | -3.94e$^{-03}$ |
| $K_p$ | t Ratio | 6.43 | 0.39 | -0.2 | -1 | 0.04 | 0.2 | 0.09 | -1.31 | -2.22 | -1.62 |
| | Prob >\|t\| | **0.0013**** | 0.7136 | 0.8493 | 0.3616 | 0.9712 | 0.851 | 0.935 | 0.2471 | 0.0767 | 0.1656 |
| | **Coefficient value** | 1.76424 | 0.02716 | 0.05 | -0.025 | -0.05 | 0.04753 | -3.463$^{-18}$ | 0.01667 | -0.03333 | 0.01910 |

| | | | | | | | | | | | |
|---|---|---|---|---|---|---|---|---|---|---|---|
| $Q_{6h}$ | t Ratio | 28.31 | 0.71 | 1.31 | -0.66 | -0.93 | 0.89 | 0 | 0.3 | -0.6 | 0.34 |
| | Prob >\|t\| | **<0.0001*** ** | 0.507 | 0.2456 | 0.5391 | 0.3942 | 0.4154 | 1 | 0.7774 | 0.5766 | 0.7488 |
| $Q_{24h}$ | **Coefficient value** | 6.29618 | 0.62449 | -0.12449 | -0.5 | 0.25 | -0.01132 | 0.01132 | -0.79167 | -1.29167 | -1.00451 |
| | t Ratio | 6.28 | 1.02 | -0.2 | -0.82 | 0.29 | -0.01 | 0.01 | -0.88 | -1.44 | -1.11 |
| | Prob >\|t\| | **0.0015** ** | 0.3543 | 0.8468 | 0.4501 | 0.7838 | 0.99 | 0.99 | 0.4188 | 0.2102 | 0.319 |
| $Q_{48h}$ | **Coefficient value** | 25.84479 | 2.76132 | -0.76337 | -2.25 | 0.25 | 0.24897 | -0.29424 | -5.125 | -6.625 | -5.96979 |
| | t Ratio | 5.55 | 0.97 | -0.27 | -0.79 | 0.06 | 0.06 | -0.07 | -1.23 | -1.59 | -1.42 |
| | Prob >\|t\| | **0.0026** ** | 0.376 | 0.799 | 0.4637 | 0.9527 | 0.9528 | 0.9443 | 0.2743 | 0.1735 | 0.2162 |
| Assay | **Coefficient value** | 109.23 | 1.62426 | -4.51307 | -1.16 | -11.08250 | 11.36870 | -8.09745 | -8.07375 | 2.29625 | -5.50378 |
| | t Ratio | 21.33 | 0.52 | -1.44 | -0.37 | -2.51 | 2.58 | -1.84 | -1.76 | 0.5 | -1.19 |
| | Prob>\|t\| | **<0.0001*** ** | 0.6253 | 0.2081 | 0.7251 | 0.0536 | 0.0492* | 0.1251 | 0.1388 | 0.638 | 0.2884 |
| pH | **Coefficient value** | 6.69280 | 0.04441 | $3.60e^{-04}$ | 0.03 | 0.0325 | 0.01460 | 0.03542 | -0.02875 | $-3.75e^{-03}$ | $-9.05e^{-03}$ |
| | t Ratio | 476.18 | 5.18 | 0.04 | 3.51 | 2.69 | 1.21 | 2.93 | -2.28 | -0.3 | -0.71 |
| | Prob >\|t\| | **0.0035** ** | **0.0035** ** | 0.9681 | **0.0172*** | **0.0435*** | 0.2808 | **0.0325*** | 0.0713 | 0.7779 | 0.5087 |
| Instability index | **Coefficient value** | 0.12260 | -0.10978 | 0.02638 | -0.01225 | $8.75e^{-03}$ | $-3.38e^{-03}$ | -0.01081 | 0.05154 | -0.01046 | $-5.64e^{-03}$ |
| | t Ratio | 10.51 | -15.43 | 3.71 | -1.73 | 0.87 | -0.34 | -1.08 | 4.93 | -1 | -0.53 |
| | Prob >\|t\| | **0.0001** ** | **<0.0001*** ** | **0.0139*** | 0.145 | 0.4234 | 0.7493 | 0.33 | **0.0044** ** | 0.3629 | 0.616 |
| Sedimentation rate | **Coefficient value** | 0.11150 | $5.57e^{-03}$ | -0.01075 | -0.03545 | 0.04355 | -0.01144 | 0.03656 | 0.01143 | 0.03350 | -0.02645 |
| | t Ratio | 4.16 | 0.34 | -0.66 | -2.17 | 1.89 | -0.5 | 1.59 | 0.48 | 1.4 | -1.09 |
| | Prob >\|t\| | **0.0088** ** | 0.7472 | 0.5396 | 0.0817 | 0.1176 | 0.6402 | 0.173 | 0.6541 | 0.2216 | 0.3252 |
| Creaming rate | **Coefficient value** | 0.05321 | -0.16185 | 0.0297104 | $-7.13e^{-03}$ | -0.02299 | $-5.97e^{-03}$ | -0.01407 | 0.12435 | -0.01906 | 0.01752 |
| | t Ratio | 3.24 | -16.15 | 2.96 | -0.71 | -1.62 | -0.42 | -1 | 8.44 | -1.29 | 1.18 |
| | Prob >\|t\| | **0.0230*** | **<0.0001*** ** | **0.0314*** | 0.5078 | 0.1651 | 0.69 | 0.3647 | **0.0004** ** | 0.252 | 0.292 |

Signif. codes: 0 ´***`0.001´**`0.01 ´*`0.05
